# Effect of probiotic containing lactobacillus, bifidobacterium and streptococcus thermophilus in critically ill patients

**DOI:** 10.1186/cc12184

**Published:** 2013-03-19

**Authors:** M Ebrahimi Mamaghani, S Sanaie, A Mahmoudpour, H Hamishehkar

**Affiliations:** 1 Tabriz University of Medical Sciences, Tabriz, Iran

## Introduction

Sepsis is the most common cause of death in ICUs [1]. Destruction of intestinal barrier function and increased translocation of bacteria to systemic blood flow can lead to sepsis [2]. Probiotics may have beneficial effects in improvement of critically ill patients by modulating intestinal barrier and reduction of inflammation [3]. The aim of this trial was to determine the effect of probiotic on inflammatory biomarkers and mortality rate of sepsis in critically ill patients in the ICU.

## Methods

This double-blind, randomized controlled trial was conducted on 40 critically ill patients admitted to the ICU. They were randomly assigned to receive placebo or probiotic for 7 days. The APACHE score, Sequential Organ Failure Assessment (SOFA) and systemic concentrations of IL-6, procalcitonin (PCT) and protein C were measured before initiation of the study and on days 4 and 7. Also, 28day mortality was evaluated for each patient.

## Results

IL-6 and PCT levels decreased and protein C levels increased significantly in probiotic group over the treatment period (*P <*0.001). There was a significant difference in IL-6, PCT and protein C levels of the 7th day between two groups (*P *= 0.001, 0.006 and <0.001, respectively). Compared with controls, probiotic was effective in improving APACHE and SOFA scores in 7 days (*P <*0.001). There was significant difference between the probiotic and control group in the 28-day mortality rate (20% vs. 55% respectively, *P *= 0.048).

## Conclusion

Probiotics reduce inflammation and mortality rate in critically ill patients and might be considered as an adjunctive therapy to sepsis.
